# Recent evolution of alternative reproductive modes in the 'living fossil' *Triops cancriformis*

**DOI:** 10.1186/1471-2148-7-161

**Published:** 2007-09-13

**Authors:** Thorid Zierold, Bernd Hanfling, Africa Gómez

**Affiliations:** 1 Department of Biological Sciences, University of Hull, Hull, HU6 7RX, UK; 2 Institut für Biowissenschaften, Technische Universität Bergakademie Freiberg, Leipziger Strasse 29, D-09599 Freiberg, Germany; 3 Museum für Naturkunde Chemnitz, DAStietz, Moritzstrasse 20, D-09111 Chemnitz, Germany

## Abstract

**Background:**

The Notostraca is a small but ancient crustacean order with a contrasting combination of a conservative morphology and a wide range of reproductive modes. The tadpole shrimp *Triops cancriformis*, includes bisexual – the putatively ancestral state -, androdioecious and hermaphrodite populations. As hermaphroditism and androdioecy confer a colonisation advantage, we expect the postglacial colonisation of northern Europe to have been effected by lineages with such reproductive modes. Therefore, N European populations should be composed of closely related lineages reflecting a recent range expansion. In contrast, glacial refugia in the south should contain bisexual populations with high haplotype diversity and more population structuring. To test these hypotheses, we analysed the geographic distribution of reproductive modes based on new and published sex ratio data. In addition, we investigated the European phylogeography of *T. cancriformis *by sequencing over a 1000 bp of mitochondrial DNA (mtDNA) in individuals from a large sample of populations of the three recognised subspecies.

**Results:**

Bisexual populations were only found in the Iberian Peninsula, with the rest of European populations showing low male proportions or no males. Androdioecious populations were found in Central and Eastern Europe. Regarding mtDNA diversity, Spanish and Moroccan populations of *T. c. mauritanicus *were highly divergent, and showed strong population structure. In contrast, *Triops c. cancriformis *and *T. c. simplex *formed a single mtDNA lineage with low haplotype diversity. This diversity was structured into two phylogenetic clades (A, B), coexisting in E Germany. Basal haplotypes of both lineages were found in the Iberian Peninsula. Most of the populations in clade A and B are either hermaphroditic or androdioecious, with the only bisexual population in these clades found in the Iberian Peninsula. The genetic divergence between these two clades suggests a split in the Late Pleistocene and their geographic distribution reflects a complex evolutionary history of European *Triops *populations, with possibly two episodes of range expansions – one of them by clade A – involving androdioecious and hermaphroditic populations.

**Conclusion:**

As we predicted, N European populations of *T. cancriformis *are closely related, with few widely distributed haplotypes and indications of a recent range expansion involving hermaphroditic/androdioecious lineages. A possible second range expansion or long distance colonisation may have created the secondary contact zone between *T. c. cancriformis/simplex *clades A and B. The large haplotype diversity and strong genetic subdivision in the Iberian Peninsula, which is known to contain only bisexual populations, strongly suggest that this area was a Pleistocene refugium for *T. cancriformis*, although the occurrence of additional eastern refugia cannot be ruled out. Our data support the status of *T. c. mauritanicus *as a separate species and the colonisation of N Africa from the Iberian Peninsula. We suggest that hermaphroditism/androdioecy has evolved recently in *T. cancriformis *and has facilitated the postglacial colonisation of northern Europe.

## Background

The widespread occurrence of outcrossing sexual reproduction has puzzled evolutionary biologists since Darwin, as reproduction either through self-fertilisation or parthenogenesis provides immediate advantages [1,2]. One of such key advantages is evident during the colonisation process [3,4], as a single selfing hermaphrodite or parthenogenetic individual can found a new population. Organisms with mixed reproductive strategies allow a unique direct comparison of the relative advantages of different reproductive modes [5,6]. Mixed strategies often have a geographic component (e.g. 'geographical parthenogenesis') which reflects the interplay between historical and selective factors [6]. The process of recolonisation of northern areas during interglacial periods of the Pleistocene might have favoured passively dispersing parthenogens or selfing hermaphrodites. As a consequence, in temperate organisms with mixed reproductive strategies, hermaphroditism is often found in areas previously covered by glaciers or permafrost and therefore, recolonisation must have taken place after the last glacial maximum. Despite this, ecological correlates have been intensively sought to explain such geographic patterns adaptively [5,7], whereas the phylogeographic context of mixed reproductive patterns has only recently begun to be investigated [8-10].

The Eurasian tadpole shrimp *Triops cancriformis *(Crustacea: Branchiopoda: Notostraca) has a mixed reproductive strategy which includes hermaphroditism, androdioecy (consisting of hermaphrodites and a small proportion of males) and bisexuality [11,12], the latter being considered to be the ancestral condition [13]. The currently recognized subspecies *T. c. simplex *(N Africa and NE Iberian Peninsula) and *T. c. mauritanicus *(NW Morocco and SW Iberian Peninsula) are considered to be bisexual, with equal or male biased sex ratios [14,15]. The nominal subspecies *T. c. cancriformis*, occurring in central and northern Europe and a disjunct area in Japan, includes hermaphrodite and androdioecious populations [16,17]. The reproductive strategy in *T. c. cancriformis *populations is controversial, although there is evidence to suggest that 'females' are in fact hermaphrodites as they can reproduce in isolation [18] and ovotestes are present [12]. However, females from a German *T. c. cancriformis *population apparently lacked testicular tissue [19] suggesting that parthenogenesis – or a mixture of bisexual and hermaphroditic reproduction in some populations – cannot be ruled out. Our preliminary microsatellite data [20] show significant heterozygote deficiencies in many populations, and genotyping of individuals reared in isolation and their offspring supports hermaphroditism. Thus, and following Sassaman [11] we consider some *T. c. cancriformis *populations to be androdioecious, as populations consist of variable proportions of hermaphrodites and males, with some populations being made solely of selfing hermaphrodites. As in other androdioecious branchiopods, *T. c. cancriformis *hermaphrodites can reproduce either by selfing or by outcrossing with males.

*T. cancriformis *inhabits temporary freshwater ponds and all three reproductive modes lead to the production of resistant diapausing cysts, which survive in the pond sediments during drought periods. These cysts are also the means of passive dispersal by wind, water currents or downstream floods, and birds [21-23], amphibians or hoofed animals [24-26]. An indication of the vagility of such cysts is that populations occur on remote islands, and are apparently found wherever suitable habitats are available [27]. Evidence for wide notostracan distribution before the Pleistocene can be derived from its abundant fossil record dating back to the Carboniferous or possibly up to the Devonian period [28]. In fact, the striking morphological similarity of some Upper Triassic *Triops *sp. fossils from Germany and extant *T. cancriformis *[29] makes this notostracan one of the best examples of morphological stasis and 'living fossils' [30]. Thus, the potentially high dispersal abilities of their diapausing cysts and the possibility of hermaphrodite reproduction could account for the wide distribution of *T. cancriformis*.

Here we present a phylogeographic analysis of European *T. cancriformis *in the context of its reproductive mode variation. So far, attempts at investigating the genetic variability of *T. cancriformis *have been hampered by its low genetic variability [31,32], therefore our phylogeographic survey of *T. cancriformis *is based on sequence variation on over 1000 bp of mitochondrial DNA containing highly informative mitochondrial genes. We hypothesize, in agreement with Longhurst [18], that hermaphrodite or androdioecious populations should occur in areas unsuited for the species during Pleistocene glacial maxima (either covered with ice sheets or permafrost), and that therefore must have been recently colonised. Further, N European populations should be composed of one or few closely related lineages reflecting a recent range expansion of hermaphroditism and/or androdioecy. In contrast, glacial refugia in S Europe should contain bisexual populations with high haplotype diversity and high population structuring. To test these hypotheses, we analysed the geographic distribution of inferred reproductive modes based on reviewed and re-analysed data concerning sex-ratio in this species and new critical data of our own. In addition, we screened a large sample of populations of the three recognised subspecies for nucleotide sequence variation in two mtDNA genes (COI and ATPase). The phylogeographic pattern is interpreted in the context of the inferred population reproductive mode. Our results provide insights into the evolution of reproductive mode and population diversification in Triops.

## Results

### mtDNA haplotype diversity

**Figure 1:**
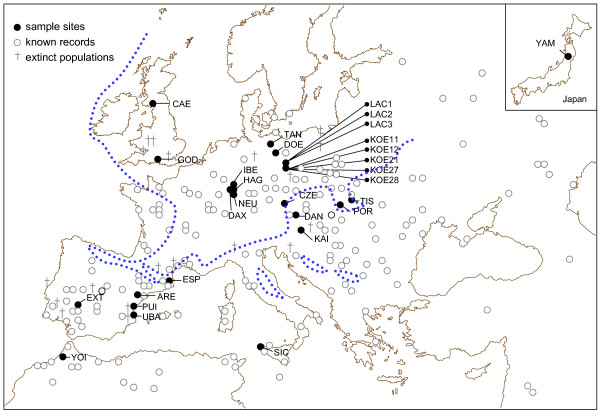
European distribution of *Triops cancriformis *showing the location of sampled populations. The dotted line indicates the continuous permafrost in the last glacial maximum (50 000–12 000 years ago) [74].

A total of 84 *T. cancriformis *individuals from 29 ponds were sequenced for two mitochondrial gene fragments: a 568 bp sequence of the cytochrome *c *oxidase 1 gene (COI) and a 506 bp fragment comprising partial sequences of ATP synthase F_O _subunits 8 (ATP8) and 6 (ATP6) genes (Table 1, Figure 1). Given that the partition-homogeneity test did not find significant differences between both fragments (p-value of 0.07), we combined them into a single alignment with a total length of 1074 bp for phylogenetic analysis.

**Table 1:** *Triops cancriformis *sample locations, population code, location of inferred subspecies, source of material, number of individuals sequenced for COI and ATPase and observed haplotypes (and numbers individuals per haplotype) per population, geographic are given based on the combined COI and ATPase analysis.

**Location**	**Code**	**Geographic location**	**Species**	**Material**	**n**	**Haplotype**
Caervalerock, UK	CAE	54°08'27" N, 003°30'58" E	Tcc	p	1	H6 (1)
Tannen, Germany	TAN	53°34'10" N, 011°25'34" E	Tcc	p	1	H2 (1)
Döberitzer Heide, Germany	DOE	52°31'43" N, 013°00'05" E	Tcc	h	2	H1 (2)
Lacoma, Germany	LAC1	51°48'12" N, 014°24'02" E	Tcc	h	2	H2 (2)
Lacoma, Germany	LAC2	51°48'05" N, 014°23'37" E	Tcc	h	3	H2 (3)
Lacoma, Germany	LAC3	51°47'47" N, 014°23'31" E	Tcc	h	2	H2 (2)
Königswartha, Germany	KOE12	51°19'50" N, 014°18'30" E	Tcc	h	3	H1 (1)
						H2 (2)
Königswartha, Germany	KOE11	51°19'49" N, 014°18'31" E	Tcc	h	3	H13 (3)
Königswartha, Germany	KOE28	51°19'48" N, 014°18'12" E	Tcc	p	4	H13 (4)
Königswartha, Germany	KOE27	51°19'46" N, 014°18'14" E	Tcc	p	3	H13 (3)
Königswartha, Germany	KOE21	51°19'43" N, 014°18'23" E	Tcc	h	9	H1 (1)
						H2 (3)
						H5 (1)
						H13 (4)
Godshill pond, UK	GOD	50°55'37" N, 001°46'30" E	Tcc	h	8	H3 (8)
Ibersheim, Germany	IBE	49°43'41" N, 008°25'55" E	Tcc	h	2	H3 (2)
Daxlander Au, Germany	DAX	49°01'10" N, 008°17'00" E	Tcc	c	2	H1 (1)
						H8 (1)
Hagenbach, Germany	HAG	49°00'40" N, 008°16'25" E	Tcc	h	1	H8 (1)
Neuburg, Germany	NEU	48°59'30" N, 008°16'25" E	Tcc	h	3	H3 (3)
Morava, Czech Republic	CZE	n.d.	Tcc	h	1	H2 (1)
Danube, Austria	DAN	n.d.	Tcc	c	2	H14 (2)
Tiszabercel, Hungary	TIZ	48°10'38" N, 021°37'18" E	Tcc	p	1	H2 (1)
Kaiserlacke, Austria	KAI	47°47'40" N, 016°52'31" E	Tcc	h	3	H2 (3)
Poroszlo, Hungary	POR	47°39'01" N, 020°43'37" E	Tcc	p	1	H7 (1)
Espolla, Spain	ESP	42°09'02" N, 002°45'60" E	Tcs*	p	7	H1 (6)
						H4 (1)
Ares del Maestre, Spain	ARE	40°25'19" N, 000°04'13" W	Tcm*	p	3	H16 (3)
El Puig, Spain	PUI	39°34'24" N, 000°16'43' E	Tcs*	p	3	H1 (3)
Laguna de la Gitanilla, Spain	EXT	39°27'00" N, 006°15'54" W	Tcm*	p	1	H15 (1)
Ullal de Baldovi, Spain	UBA	39°14'55" N, 000°19'03" E	Tcs	p	2	H11 (1)
						H12 (1)
Gorgo di Baglio Cofano Sicily, Italy	SIC	38°06'11" N, 012°40'39" E	Tcc	p	2	H10 (2)
Yamagata Prefercture, Japan	YAM	38°02'58" N, 140°10'48" E	Tcc	p	4	H9 (4)
Youssofia, Morocco	YOI	32°17'06" N, 008°19'22" W	Tcm	p	5	H17 (2)
						H18 (2)
						H19 (1)

p, preserved individuals collected in the wild; h, preserved individuals from laboratory rearing; c, cyst

Overall 213 variable nucleotide sites, 158 of them parsimony informative, were identified in the combined alignment. Excluding the Iberian (Extremadura, EXT; Ares del Maestre, ARE) and Moroccan (Youssofia, YOI) *T. c. mauritanicus *specimens, 38 sites remained variable and 26 sites were parsimony informative. Comparing the COI and ATPase (including ATP8 and 6 genes) fragments separately we found within the COI fragment a total of 20 substitutions on 1^st ^codon position, 3 on 2^nd ^and 91 on 3^rd ^positions, which resulted in nine amino acid changes. The ATPase fragment is characterised by 46 changes on 1^st ^codon position, 22 on 2^nd ^and 99 at 3^rd ^codon position which result in 33 amino acid changes. The combined alignment was moderately A+T rich (mean AT content = 65.2%).

**Figure 2:**
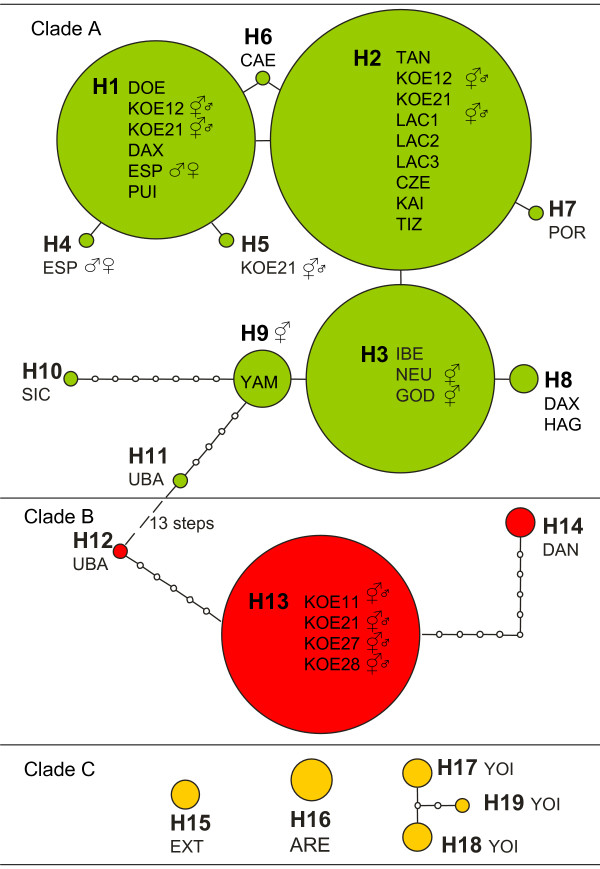
Maximum parsimony network of *Triops cancriformis *based on combined COI and ATPase sequence data. Each circle represents a different haplotype, with its diameter proportional to the haplotype sample size. Each line between haplotypes represents a mutation. Populations containing each haplotype and their reproductive mode (if known) are indicated. Reproductive mode symbols as in Figure 3. Haplotypes of *T. c. mauritanicus *could not be joined to the *T. c. cancriformis/simplex *network or between themselves without exceeding the limits of parsimony, and form three disjointed networks.

Despite sequencing over 1000 bp of mtDNA, overall haplotype diversity was low, with only 19 mtDNA haplotypes identified in the combined alignment. Eleven of those haplotypes were found in the 14 *T. c. cancriformis *populations sampled. Fourteen out of the nineteen identified haplotypes were found in single populations (Table 1, Figure 2), while one haplotype (H8) was found in two nearby populations in Germany (DAX, HAG), H13 was found in a set of four nearby ponds in the area of Königswartha, and H1, H2 and H3 were common and widespread, found in six, nine and three ponds respectively. H1 was found in the Iberian individuals from Espolla (ESP) and El Puig (PUI), which had been previously identified as *T. c. simplex *[33], as well as in individuals of the nominal subspecies from Döberitzer Heide (DOE), Königswartha pond 12 and 21 (KOE12, KOE21) and Rhine (DAX).

The number of haplotypes per population ranged from 1–4 (average 1.35), with the maximum found in Königswartha (KOE21) (Table 1). Given the low level of genetic diversity found in our preliminary analysis, we optimised our resources by increasing the number of bp sequenced per individual and the number of populations surveyed, instead of the number of individuals sequenced per population.

### Phylogenetic relationships

**Figure 3:**
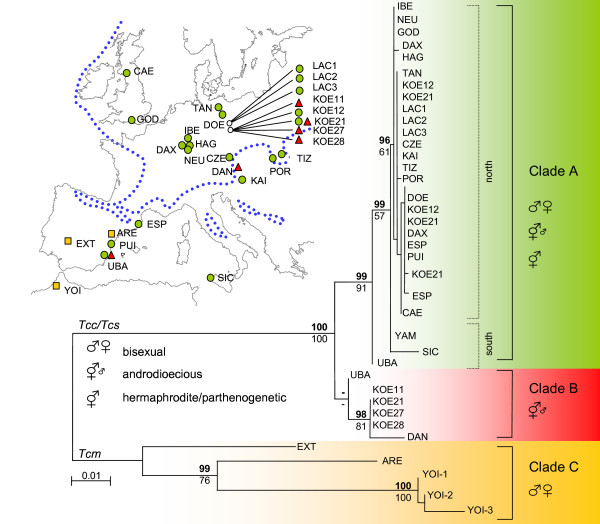
Maximum likelihood (ML) tree obtained from the combined COI and ATPase sequence alignment and the distribution of COI-ATP *Triops cancriformis *haplotypes in Europe (Tcc/s, *Triops cancriformis cancriformis/simplex*; Tcm, *Triops cancriformis mauritanicus*). In the phylogram, values above major branches are posterior probabilities in the Bayesian analysis; numbers below indicate the bootstrap support in the Maximum likelihood analysis (only values over 50% are shown). The tree is midpoint rooted. Inferred reproductive mode in populations of each clade is indicated by symbols. In the different clades are represented by coloured symbols in the map (green circles for clade A; red triangles for clade B; yellow squares for clade C).

Both maximum likelihood and Bayesian analyses produced identical topologies showing two highly divergent and strongly supported monophyletic lineages (Figure 3). The first lineage is represented by *T. c. mauritanicu*s haplotypes (nucleotide diversity π = 0.05905) (clade C). The second lineage includes all haplotypes from the subspecies *T. c. cancriformis *and *T. c. simplex *(π = 0.00920). Despite the low genetic diversity within *T. c. cancriformis/simplex*, two divergent clades (A and B) were identified. The closely related haplotypes H1 to H9 within clade A occurred from E Spain to N and C Europe and the Japanese population (π = 0.00114) (see network in Figure 2). This group includes the three most common and widespread haplotypes (H1, H2, H3) and a cluster of rare, geographically restricted haplotypes differing from them in single substitutions. In contrast, haplotypes from the southern populations within clade A (H10, Sicily and H11, UBA in Spain) are more divergent and differ in 9 and 6 respectively substitutions from H3. The two morphologically described subspecies *T. c. cancriformis *and *T. c. simplex*, which show different reproductive modes, are not represented by distinct monophyletic lineages, as *T. c. simplex *individuals from some East Iberian populations (PUI, ESP) share haplotype H1 with five *T. c. cancriformis *individuals from four different populations in Germany. Clade B was only found in three disjunct locations (KOE, DAN, UBA, see Figure 3). Haplotypes from clades A and B were present in Königswartha populations. Population UBA from eastern Spain contained the basal haplotype of clade A (H11) – the most internal haplotype in the network – and a haplotype of unresolved position (H12) according to the phylogenetic analyses, but which appears as most closely related to clade B from our network analysis under the limits of parsimony (Figure 2), and therefore was included in clade B. The root of the *T. c. cancriformis/simplex *lineage lies in between both haplotypes in this population, which seems to have retained ancestral polymorphisms in the species.

### Sequence divergence and approximation of divergence times

Based on the combined alignment, pair-wise nucleotide differences among all *T. cancriformis *haplotypes ranged from 1 to 137 bp which translate into a maximum distance (HKY + G corrected) of 14.38% (H5 vs. H17). The mean corrected distances between clades for the combined alignment and for the COI and ATPase fragments separately are shown in Table 2. The genetic distance between clades A and B, including the two recognized subspecies *T. c. cancriformis *and *T. c. simplex*, is less than 3.5%. The mean genetic distance was considerable higher (>10%) between haplotypes from clade A and B together (*T. c. cancriformis *and *T. c. simplex*) and clade C (*T. c. mauritanicus*).

**Table 2:** Mean corrected percent sequence divergences between *Triops cancriformis *clade/population comparisons and estimated divergence times based on corrected COI distance. Distances are given for the combined COI-ATPase fragment as well as separately for COI and ATPase sequences. Because no crustacean molecular clock calibration factors are available for ATPase we used the COI corrected distance for approximations of divergence times. For population codes see Table 1, for clade definition see Figure 2.

	**Corrected % sequence divergence**			**Estimated divergence times (mya)**	
		
**clade/population**	**COI-ATP**	**ATP**	**COI**	**1.4% sequence divergence per mya**	**2.3% sequence divergence per mya**
clade A vs. clade B	2.1590	3.4323	1.5865	1.1332	0.6809
clade A and B vs. C	13.5477	44.5345	10.985	7.8465	4.7146
ARE vs. YOI	9.7768	24.3929	7.9947	5.6762	3.4106
ARE&YOI vs. EXT	10.4396	31.5413	7.8278	5.5912	3.3595

Calculating the corrected distances separately for the COI and ATPase fragment resulted in higher values for the ATPase alignment. This illustrates, that ATPase is evolving faster and provides more information regarding intraspecific relationships than COI (Table 2).

The crustacean COI calibration applied to obtain an approximate time of divergence between the *T. c. cancriformis*/*simplex *group (clades A and B) and *T. c. mauritanicus *(clade C), yielded 4.71–7.85 mya (around the Miocene-Pliocene boundary) (Table 2). The divergence time between clades A and B was estimated to have happened during the Early Pleistocene (0.68–1.13 mya).

### Distribution of inferred reproductive modes in Triops cancriformis

**Figure 4:**
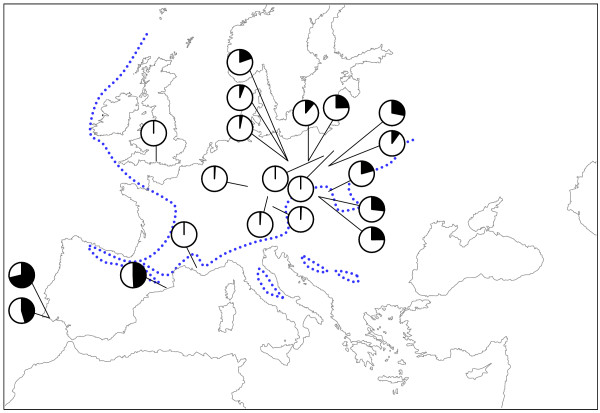
Geographic variation of male proportions in *Triops cancriformis *(for data and references see Table 3). The pie charts show the proportion of males in black for each population with sample size over 25. The dotted line indicates the maximal extent of permafrost in the last glacial maximum [74].

Bisexuality was supported in two Portuguese populations of *T. c. mauritanicus *(Algarve) and one Spanish population of *T. c. simplex *(Espolla) (Table 3, Figure 4). Androdioecy was inferred for populations in Central and E Europe (Table 3). Populations with male proportions above 0.100 were identified mainly from eastern European populations (Poland and Hungary). As listed in Table 3, male-less populations occur in Germany, France, Poland and United Kingdom, and no males have been reported from Italian, Polish and Japanese populations (with no reported sample sizes) [17,34-36]. As androdioecious populations can have low proportion of males it can not be excluded that those population are actually androdioecious. In populations where males have been recorded male proportions ranged from 0.012 to 0.281 (Table 3). As shown in Figure 4 both androdioecious and putatively hermaphrodite populations are distributed where no suitable habitats for *T. cancriformis *were found during the Pleistocene ice ages.

**Table 3:** Sex ratio and inferred reproductive mode in European *Triops cancriformis*. The number (n) of individual males and females/hermaphrodites and the male proportion per pond are given. The results of the Chi-Square test to infer deviations from a null hypothesis of equal sex ratio and the inferred type of reproductive mode are provided (see text). In the populations investigated here, the collection year is indicated under the Reference heading.

**Location**	**n_male_**	**n_female/hermaphrodite_**	**prop. of males**	**p value (Chi-Square)**	**Inferred reproductive mode**	**Reference**
Baillargues, France	0	200	0.000	<0.0001	hermaphrodite	[35]
Neuburg, Germany	0	102	0.000	<0.0001	hermaphrodite	own data (2004–2006)
Jaktorów, Poland	0	40	0.000	<0.0001	hermaphrodite	[75]
Zabieniec, Poland	0	40	0.000	<0.0001	hermaphrodite	[75]
Godshill pond, United Kingdom	0	25	0.000	<0.0001	hermaphrodite	this paper (2004–2006)
Königswartha (KOE25), Germany	2	32	0.059	<0.0001	androdioecious	this paper (2006)
Bavaria, Germany	8	1000	0.008	<0.0001	androdioecious	[76]
Augsburg, Germany	7	568	0.012	<0.0001	androdioecious	[77]
Königswartha (KOE12), Germany	10	95	0.095	<0.0001	androdioecious	this paper (2004 to 2006)
Cracow, Poland	16	144	0.100	<0.0001	androdioecious	[78]
Königswartha (KOE27), Germany	14	123	0.102	<0.0001	androdioecious	this paper (2006)
Wroclaw, Poland	114	912	0.111	<0.0001	androdioecious	[79]
unknown location, Hungary	9	34	0.209	0.0001	androdioecious	[80]
Wroclaw, Poland	29	88	0.248	<0.0001	androdioecious	[79]
Lake Balaton, Hungary	15	45	0.250	0.0001	androdioecious	[81]
Lake Balaton, Hungary	7	19	0.269	0.0186	androdioecious	[81]
Cracow, Poland	154	395	0.281	<0.0001	androdioecious	[78]
Algarve, Portugal	19	23	0.452	0.5371	bisexual	[82]
Algarve, Portugal	54	22	0.711	0.0002	bisexual	[82]
Espolla, Spain	64	57	0.529	0.5245	bisexual	this paper (2006)
Espolla, Spain	1723	1775	0.493	0.3872	bisexual	[33]

## Discussion

As we predicted, *T. cancriformis *haplotypes from most N European populations and the disjunct Japanese population – clustered in clade A – are closely related, with a few, widely distributed haplotypes. The reduced genetic diversity in *T. cancriformis *is a consistent and remarkable result of our data. This can be supported by the large mitochondrial fragment we investigated, the selection of the most rapid evolving gene in *Triops *and extensive sampling. A low mitochondrial diversity in *T. cancriformis *was noted by Mantovani et al. [32] and Korn et al. [31] based on 16S and 12S sequences, which are slowly evolving compared to our fragments. Furthermore, preliminary data on diversity of nuclear microsatellite loci [20,37] suggested low genome wide polymorphism in *T. c. cancriformis/simplex *[20]. This pattern supports postglacial range expansion of *T. c. cancriformis/simplex *lineages into areas which were unsuitable for this species during the last glacial maximum. When we take the phylogeographic and reproductive mode results together, the predominance of androdioecious and potentially hermaphroditic populations in formerly glacial or permafrost regions strongly suggests that lineages with alternative reproductive modes were responsible for the postglacial recolonization of N Europe, as hypothesized by Longhurst [18]. Our results with *T. cancriformis *closely resemble the pattern found in geographic parthenogenesis. The term geographical parthenogenesis is used for the tendency of parthenogenetic populations to be distributed in high latitudes reflecting an association between parthenogenesis and environments that were strongly affected by the Pleistocene glacial cycles [6,38]. Several organisms have been described having parthenogenetic populations in N and C Europe and sexual populations in restricted southern areas [39,40]. Although *T. cancriformis *populations, being hermaphroditic, do not fulfil the geographical parthenogenesis concept, they would enjoy the colonisation advantage in a comparable way. Therefore we suggest broadening the concept of geographical parthenogenesis by introducing the term 'geographical hermaphroditism'.

In contrast to the pattern found in N Europe with populations of low genetic diversity and alternative reproductive modes, southern *T. cancriformis *populations, including all the identified bisexual populations, show high and geographically structured haplotype diversity. These results support the hypothesis that bisexual populations are characterised by reduced gene flow and high interpopulation divergence (regional differentiation as found between Iberian *T. c. mauritanicus *populations), possibly because the areas inhabited by these populations have been suitable for longer. Similar strong geographic structuring of genetic variation with very isolated populations has been reported from other sexual European large branchiopods (Anostracans) [41,42]. These strong population structures despite high potential for gene flow through their diapausing propagules suggests that *Triops *populations and other large branchiopods, in a similar way to continental zooplankton undergo a process of 'Monopolisation' of their habitats [43]. The presence of ancestral haplotypes and strong genetic subdivision in the Iberian Peninsula, strongly points to this area as a Pleistocene refugium for *T. cancriformis*. This inference is further supported by the presence of bisexual populations for *T. c. mauritanicus *and *T. c. simplex *in the Iberian Peninsula. However, the genetic divergence between clades A and B and their geographic distribution indicates that the phylogeographic history of European *T. cancriformis *is more complex as these clades are likely to have diverged earlier than the last glacial cycle. Clade B is especially puzzling as it is present only in three locations including northern and southern populations. Different scenarios could explain this pattern. One of these is the possibility that N European populations from clade B are derived from an early postglacial expansion which left relict populations in an Eastern refugium – Balkans, Middle East – which we did not sample, a pattern that has previously been reported for the European fire salamander [44]. Another explanation could be long distance colonisation events facilitated by the passive dispersal of *Triops *diapausing cysts as reported for other aquatic invertebrates [45,46]. Either way, this possible range expansion/long distance colonisation event – comparable to clade A – was linked to androdioecious lineages, which is the reproductive mode, present in clade B. In summary we suggest a split in the Late Pleistocene that originated clades A and B in *T. c. cancriformis/simplex*, with an early refugium in the Iberian Peninsula and possibly a second one. Further sampling from eastern populations and further Mediterranean islands where the species is known to occur should shed light into this issue. Subsequently these lineages underwent possibly two episodes of range expansions/long distance colonisation, in both cases involving hermaphroditic and/or androdioecious populations. Interestingly, those populations we infer to be androdioecious, occur in the area where both clades coexist in Germany. The occurrence of hybridisation between these clades is suggested by our microsatellite data [20] as two of the populations where both lineages coexist (KOE) and have been analysed with microsatellite loci display Hardy Weinberg equilibrium [20].

We suggest that geographic isolation within the Iberian Peninsula, possibly dating back to the Miocene-Pliocene boundary, led to the initial divergence between *T. c. mauritanicus *and *T. c. cancriformis/simplex *lineages and also to genetic isolation among *T. c. mauritanicus *populations in the Northwest and Southeast Iberian Peninsula. Such regional persistence suggests that long-term suitable habitats were available in separate areas within the Iberian Peninsula throughout the Pliocene and Pleistocene. Deep phylogeographic splits within the Iberian Peninsula have been observed in a wide range of taxa, indicating either the existence of several independent Pleistocene refugia within this area [47] or colonisation of the southern Iberian Peninsula from North Africa [48,49]. Our analyses, containing Moroccan samples, indicate that the Iberian populations of *T. c. mauritanicus *are basal, which supports a scenario in which *T. c. mauritanicus *would have colonised North Africa from the Iberian Peninsula, possibly during the early Pliocene. A similar pattern of colonisation from the Iberian Peninsula into North Africa has been documented for lizards and amphibians [50-52]. Therefore, we suggest that the Iberian Peninsula provided a long-term subdivided glacial refugium for both *T. c. mauritanicus *and *T. c. cancriformis/simplex*.

The combined alignment of COI and ATPase sequences showed that *T. c. mauritanicus *and *T. c. cancriformis/simplex *form two highly divergent monophyletic lineages, as also suggested by a 16S and 12S analysis [31]. The estimated 13.55% sequence divergence between both lineages is within the range separating other notostracan [53] and crustacean species [54]. Our data therefore provide strong support to the proposal of Korn et al. [31] to reinstate *T. c. mauritanicus *to species status, as originally described by Ghigi [55]. In contrast, the traditional subdivision into the subspecies *T. c. cancriformis *and *T. c. simplex sensu *Longhurst [27] could not be supported by our data as the populations representing *T. c. simplex *(ESP, PUI, UBA) shared either a genetic haplotype with *T. c. cancriformis *populations or were very closely related to them. We propose that the validity of *T. cancriformis simplex *should be reconsidered.

## Conclusion

Our analysis of *T. cancriformis *reproductive mode in the context of its phylogeography allowed us to gain insights into the role of alternative reproductive modes during Pleistocene range shifts. We suggest that hermaphroditism/androdioecy has evolved recently, possibly in the Late Pleistocene and has facilitated the postglacial colonisation of northern Europe from one or two refugia, one of them likely placed in the Iberian Peninsula. Despite generally low genetic variability in *T. cancriformis *we were able to identify mitochondrial regions which provided sufficient resolution to reveal phylogeographic patterns across Europe. Our data support a taxonomic revision of *T. cancriformis*. The occurrence of three different reproductive modes within closely related genetic lineages of a single species make *T. cancriformis *a good model to investigate the evolution of mixed reproductive strategies.

## Methods

### Samples

A total of 29 water bodies in inundated floodplains, isolated ponds, puddles and fish nursery pools containing *T. cancriformis *populations were sampled in Europe, Japan and Morocco between 2000 and 2006 (Table 1, Figure 1). Overall 23 populations belong to the nominal subspecies, *T. c. cancriformis*, 3 populations were identified as the subspecies *T. c. simplex *and 3 populations belong to *T. c. mauritanicus *(Table 1). Samples included specimens collected using a dip net (5 mm pore diameter), and sediment containing diapausing cysts from dry ponds where the species was known to occur. Once specimens were needed for genetic analyses, and to be able to obtain sex ratio data, sediment was placed at the bottom of glass tanks and distilled water added to induce hatching of cysts. Individuals were grown until maturity and fed daily with fish flakes ad libitum.

### Amplification and Sequencing

Total genomic DNA was isolated from ethanol-preserved tissue using commercial DNA extraction kits (Invisorb Spin Forensic Kit, Invitek; PureGene Kit, Gentra Systems). For the populations DAN and DAX, where attempts to hatch specimens from the sediment were unsuccessful, DNA was extracted from single cysts isolated from the sediment following procedures described in Gómez & Carvalho [56] (Table 1).

Given that our preliminary studies based on sequence variation at mitochondrial 16S and COI genes provided little resolution for intraspecific relationships within *T. cancriformis *[20] we decided to explore the published *T. cancriformis *and *T. longicaudatus *mitochondrial genomes in order to identify the most variable regions for a phylogeographic survey. We designed primers within highly conserved regions identified from an alignment of four branchiopoda sequences, *Triops cancriformis *(GenBank accession number NC_004465), *Triops longicaudatus *(NC_006079), *Daphnia pulex*, (DQ340834) and *Artemia franciscana *(X69067). The set of primers are available from the authors under request. We then carried out a preliminary survey of genetic variation for nine mitochondrial genes (ATP6, ATP8, cytb, ND2, ND3, ND5, ND4, ND4L and the D-loop) using eight *T. cancriformis *individuals from different populations. As a result ATP8 and ATP6 in combination with COI proved to be the most diverse fragments in terms haplotype and nucleotide diversity (data available from the authors upon request). Consequently these loci were chosen to complement our preliminary COI data.

A 568 bp fragment of COI was amplified and sequenced using primers LCO1490 and HCO2198 [57]. A second fragment of 506 bp, covering parts of the ATP8 and parts of the ATP6 gene, was amplified using newly designed *Triops *specific primers. The 5' primer, ATP-Tc-3800U (5'-TCTAATCCCCCAAATAGCCCCTAGT-3'), is located in the tRNA^Asp ^(corresponding to sites 3800 to 3825 in the reference sequence NC_004465), and the 3' primer, ATP-Tc-4405L (5'-ACAGCAAGAGTTCCGGGACGAATA-3') is located in the ATP6 mtDNA (sites 4381 to 4405 in the reference sequence NC_004465).

Amplifications were performed in 20 μl final volume containing 2 μl template DNA, 1.5 mM MgCl_2_, 200 μM of each nucleotide, 100 μM of each primer, 0.01 U of *Taq *DNA polymerase and 1× NH_4_-PCR Buffer (Bioline). The following cycling conditions were used: 3 min denaturating at 93°C, (45 s at 94°C, 45 s at 50°C, 1 min at 72°C) × 35, 5 min extension at 72°C. PCR products were sequenced directly using the PCR primers and the Beckman DTCS Quick Start Sequencing kit in a Beckman CEQ8000 capillary sequencer. The sequences were checked by eye with the CEQ8000 data analyser and initially aligned with Clustal W [58] and finally adjusted by hand. Sequences for the COI, ATP6 and ATP8 fragments were obtained for all individuals. All the sequences were deposited in GenBank (accession numbers EF675826–EF675991).

### Phylogenetic reconstructions

A partition-homogeneity test [59] was performed using PAUP*4.0 [60] on the COI and ATPase datasets to determine whether datasets were congruent and could be combined for phylogenetic analyses.

Phylogenetic relationships were reconstructed using maximum-likelihood and Bayesian approaches. The best fitting, least-parameter rich model of sequence evolution was based on hierarchical likelihood-ratio test in the program ModelGenerator0.6 [61]. This method identified the HKY+G model of sequence evolution and four gamma distributed rate categories as the optimal model. This model of sequence evolution and its parameter estimates were used to perform a maximum likelihood (ML) algorithm in PHYML 2.4.4 [62]. Branch support of the ML tree was assessed by 1000 bootstrap pseudo-replications. MRBAYES 3.1.1 [63] was used to generate a Bayesian reconstruction by running a Markov chain for 2,000,000 generations and using a burn-in of 2500. The 50% consensus tree was obtained from the remaining trees from both runs sampled after the initial burn-in period.

Nodes in the ML-tree were considered to be well supported if they showed at least 70% bootstrap support [64]. Similarly, strong branch support was inferred in the Bayesian analyses when posterior probabilities were over 80% [65]. Trees were displayed with NJPlot [66]. To examine the geographic and reproductive mode associations of haplotypes a haplotype network was constructed using TCS1.21 [67].

### Molecular clock estimates

Unfortunately, the abundant fossil record of Triopsidae is not informative for molecular calibration purposes mainly due to the morphological stasis of the group since the Carboniferous period (about 302 Ma) [28,68-70]. Therefore, currently, fossil data provide no information on splits within *Triops*. In consequence, no direct calibration points were available within the Triops phylogeny precluding the application of methods to estimate divergence times such as suggested by Sanderson [71]. In order to provide a rough approximation of divergence times among *T. cancriformis *lineages we used two crustacean clock calibrations of Knowlton and Weigt [72] (1.4% sequence divergence per million years) and Schubart et al. [73] (1.66–2.33% sequence divergence per million years). Both calibrations are based on COI data and consequently we used only the COI fragment from our data set to estimate divergence times. Corrected average pairwise genetic sequence (using the best fit model for the COI dataset, TVM+G) between haplotypes were used in all divergence time estimates.

### Sex ratio and reproductive mode

Sex ratio was determined for all *T. cancriformis *populations sampled in this study either from wild-caught specimens or from individuals hatched in the laboratory from sediment samples. Sexing was carried out on fixed specimens under a dissecting microscope. We considered an individual was a male when no egg sac was found in the 11^th ^pair of thoracopods. No external diagnostic character allows discriminating between females and hermaphrodites; therefore, we calculated the sex ratio as the number of males divided by the number of female/hermaphrodites. Furthermore we included published data for 21 European populations in order to understand the geographic distribution of sex ratios. We only included literature data that provided either sample sizes and the sex ratio, or the raw numbers of males and females/hermaphrodites per sample. Sex ratio was used to distinguish between three potential reproductive modes for each population: bisexual reproduction was inferred when the sex ratio did not significantly differ from 1 based on a binominal Chi-square test; androdioecy was inferred when males where present but the sex ratio deviated significantly from 1; populations in which no males where found were treated as potentially hermaphroditic populations. Note, however, that such populations could also represent androdioecy with low frequency of males.

## Authors' contributions

TZ participated in the design of the study, carried out the molecular genetic studies, sequence alignment and sex-ratio analysis and drafted the manuscript. BH participated in the design of the study and the statistical analysis and helped to draft the manuscript. AG conceived the study, and participated in its design, the analysis and interpretation of data and helped to draft and revise the manuscript it critically for important intellectual content. All authors read and approved the final manuscript.
